# Procalcitonin for diagnosis of infection and guide to antibiotic decisions: past, present and future

**DOI:** 10.1186/1741-7015-9-107

**Published:** 2011-09-22

**Authors:** Philipp Schuetz, Werner Albrich, Beat Mueller

**Affiliations:** 1 Harvard School of Public Health, 667 Huntington Ave, 02115 Boston (MA), USA; 2 University Department of Medicine, Kantonsspital Aarau, Tellstrasse, CH-5001 Aarau, Switzerland

## Abstract

There are a number of limitations to using conventional diagnostic markers for patients with clinical suspicion of infection. As a consequence, unnecessary and prolonged exposure to antimicrobial agents adversely affect patient outcomes, while inappropriate antibiotic therapy increases antibiotic resistance. A growing body of evidence supports the use of procalcitonin (PCT) to improve diagnosis of bacterial infections and to guide antibiotic therapy. For patients with upper and lower respiratory tract infection, post-operative infections and for severe sepsis patients in the intensive care unit, randomized-controlled trials have shown a benefit of using PCT algorithms to guide decisions about initiation and/or discontinuation of antibiotic therapy. For some other types of infections, observational studies have shown promising first results, but further intervention studies are needed before use of PCT in clinical routine can be recommended. The aim of this review is to summarize the current evidence for PCT in different infections and clinical settings, and discuss the reliability of this marker when used with validated diagnostic algorithms.

## Background

Emerging bacterial resistance to antimicrobial therapeutics calls for more stringent efforts to reduce antibiotic overuse [1]. Towards this aim, there has been considerable interest in antibiotic stewardship programs aimed at reducing antibiotic overuse by tailoring antibiotic therapy to individual needs of patients [2,3]. Despite the successful implementation of diagnostic biomarkers in different fields of medicine (for example, D-dimers in pulmonary embolism, natriuretic peptides in acute heart failure, troponin in myocardial infarction), accurate and timely diagnosis of bacterial infections remains a challenge [4,5]. Reliable clinical and/or microbiological parameters from easy to obtain specimens that may be used to diagnose bacterial infections and rule out other infections not in need of antibiotic therapy have been largely lacking. The main disadvantages of many current microbiological methods are diagnostic delays (for example, culture methods), suboptimal sensitivity (for example, blood cultures) and low specificity due to contamination (for example, sputum cultures), whereas others are not amenable to routine diagnostics due to their invasive nature (for example, lung biopsy). Inflammatory markers, such as C-reactive protein (CRP) or white blood cells (WBC), lack specificity for bacterial infections [6]. This is partly explained by the heterogeneity of different infections and the complex interaction of different pro- and anti-inflammatory mediators of the host response aimed at combating invading pathogens during systemic infections, which depend on timing, type, extent and site of the underlying infection.

In this diagnostic dilemma, procalcitonin (PCT) has stimulated great interest as a potentially more specific marker for bacterial infection. PCT is produced ubiquitously in response to endotoxin or mediators released in response to bacterial infections (that is, interleukin (IL)-1β, tumor necrosis factor (TNF)-α, and IL-6) and strongly correlates with extent and severity of bacterial infections [7]. Because up-regulation of PCT is attenuated by interferon (INF)-γ, a cytokine released in response to viral infections, PCT is more specific for bacterial infections and may help to distinguish bacterial infections from viral illnesses [8-11]. PCT shows a favorable kinetic profile for use as a clinical marker: it promptly increases within 6 to 12 hours upon stimulation and circulating PCT levels halve daily when the infection is controlled by the host immune system or antibiotic therapy [12]. PCT correlates with bacterial load [13-15] and severity of infection [6,16-18]. PCT thus has prognostic implications and the course of PCT predicts fatal outcome in patients with community-acquired pneumonia (CAP) [18-21] and critically ill patients with sepsis [22].

**Figure 1:**
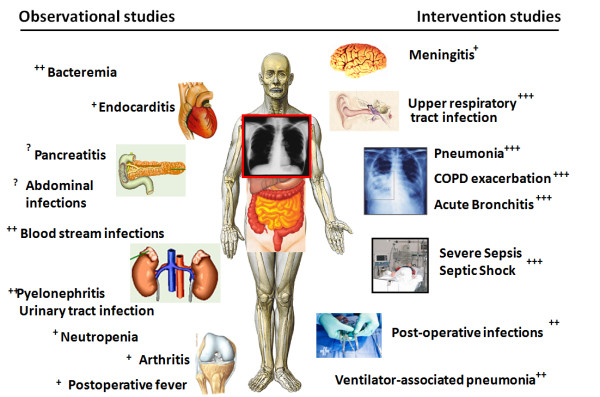
**Available evidence concerning PCT in different infections derived from observational and randomized-controlled intervention studies**. While for some infections, intervention studies have investigated benefit and harm of using PCT for antibiotic decisions (right side), for other infections only results from diagnostic (observation) studies are available with mixed results (left side). Abbreviations: PCT, procalcitonin. + moderate evidence in favor of PCT; ++ good evidence in favor of PCT; +++ strong evidence in favor of PCT; ? evidence in favor or against the use of PCT still undefined

Based on this evidence, PCT has been put forward as a promising candidate marker for diagnosis and for antibiotic stewardship in patients with systemic infections [23]. Importantly, as with any diagnostic tool, PCT should be used embedded in clinical algorithms adapted to the type of infection and the clinical context and setting. While for some types of infections and clinical settings optimal PCT cut-offs have been established and their safety and efficacy shown in randomized-controlled intervention trials, for other types of infection only observational studies are available today (Figure 1), and thus the clinical benefit and safety of using PCT remains undefined.

The aim of this review is to summarize the current evidence for PCT in different infections and clinical settings, and discuss the strengths and limitations of PCT, and the reliability of this marker when used with validated diagnostic algorithms.

## Procalcitonin as a diagnostic marker: results from observational studies

A plethora of observational studies have investigated the diagnostic potential of PCT in different clinical situations and different types and sites of infections. Table 1 summarizes study designs, proposed PCT cut-offs and main conclusions of selected relevant studies investigating different types of infections. This selection focuses on more recent research and on studies using highly sensitive PCT assays (that is, with a functional assay sensitivity around 0.06 μg/L) [24,25].

**Table 1:** Overview of studies investigating the use of PCT in different types and sites of infection

Type of infection	Study designs	PCT cut-off (ug/L)	Benefit of using PCT?	Main conclusions	Selected References
Abdominal Infections	observational	0.25	?	PCT may help to exclude ischemia and necrosis in bowel obstruction	[29-32]
Arthritis	observational	0.1-0.25	+	PCT differentiates non-infectious (gout) arthritis from true infection	[37,38,73]
Bacteremic infections	observational	0.25	++	Low PCT levels help to rule out bacteremic infections	[14,15,74]
Blood stream infection (primary)	observational	0.1	++	PCT differentiates contamination from true infection	[13]
Bronchitis	RCT	0.1-0. 5	+++	PCT reduces antibiotic exposure in the ED without adverse outcomes	[50,52]
COPD exacerbation	RCT	0.1-0. 5	+++	PCT reduces antibiotic exposure in the ED and hospital without adverse outcomes	[50-52]
Endocarditis	observational	2.3	+	PCT is an independent predictor for acute endocarditis with high diagnostic accuracy	[27,28]
Meningitis	before-after	0.5	+	PCT reduces antibiotic exposure during outbreak of viral meningitis	[75-77]
Neutropenia	observational	0.1-0.5	+	PCT is helpful at identifying neutropenic patients with systemic bacterial infection	[39-41]
Pancreatitis	observational	0.25-0.5	?	PCT correlates with severity and extend of infected pancreatitis	[33,36]
Pneumonia	RCT	0.1-0. 5; 80-90% **↓ **	+++	PCT reduces antibiotic exposure in the hospital without adverse outcomes	[16,50,52-55]
Postoperative fever	observational	0.1-0.5	+	PCT differentiates non-infectious fever from post-operative infections	[78]
Postoperative Infections	RCT	0.5-1.0; 75-85% ↓	++	PCT reduces antibiotic exposure in the surgical ICU without adverse outcomes	[64,65]
Severe sepsis/Shock	RCT	0.25-0.5; 80-90% ↓	+++	PCT reduces antibiotic exposure in the ICU without adverse outcomes	[61,62]
Upper respiratory tract infections	RCT	0.1-0.25	++	PCT reduces antibiotic exposure in primary care without adverse outcomes	[58]
Urinary tract infections	observational	0.25	+	PCT correlates with severity of urinary tract infections	[15,26]
Ventilator-associated pneumonia	RCT	0.1-0.25	++	PCT reduces antibiotic exposure without adverse outcomes	[62,63]

Abbreviations: COPD, chronic obstructive pulmonary disease; ED, Emergency department; PCT, procalcitonin; RCT, randomized-controlled trial. The level of evidence in favor or against PCT for each infection was rated by two of the coauthors (PS, WCA) independently and disagreements were resolved by consensus.

+ moderate evidence in favor of PCT; ++ good evidence in favor of PCT; +++ strong evidence in favor of PCT; ? evidence in favor or against the use of PCT still undefined

For the diagnosis of blood stream infections and bacteremia, studies found a high diagnostic performance of PCT [13-15]. To distinguish blood contamination from true blood stream infection in patients with growth of coagulase-negative staphylococci in their blood cultures, PCT demonstrated a better discriminatory ability compared to WBC and CRP [13]. At a cut-off of 0.1 ug/L, PCT had a very high sensitivity to exclude true infection. Two other studies, focused on the use of PCT to predict bacteremia infections in patients with CAP [14] and urinary tract infections (UTI) [15]. A PCT cut off of 0.25 ug/L was most helpful to exclude bacteremic disease with a high negative predictive value in both settings.

In UTIs, evidence for the utility of PCT comes primarily from the pediatric literature, where it has a similar sensitivity but superior specificity compared to CRP for the prediction of pyelonephritis in children with febrile UTIs [26]. It correlates with the extent of renal involvement and with renal scarring. Similarly, in patients with infectious endocarditis, circulating PCT levels were elevated compared to non-infected patients in two independent studies [27,28]. Unfortunately, a reliable PCT threshold for diagnosing or excluding infective endocarditis was neither proposed nor tested in intervention studies. Importantly, subacute forms of endocarditis or prosthetic-valve endocarditis may show different characteristics compared to acute forms due to their low inflammatory nature and possibly biofilm production.

Few studies have investigated the use of PCT in intra-abdominal infections [29-36]. While PCT showed promise as a marker to exclude perforation and ischemia in obstructive bowel syndrome [32], the utility in acute appendicitis [31] and pancreatitis [33,36] was limited and PCT was more helpful as a prognostic marker for severe disease and adverse outcome. While localized infections may not induce a massive PCT up-regulation, studies found PCT of diagnostic utility in patients with arthritis [37] and osteomyelitis [38], particularly when subtle increases and a low PCT cut off (0.1 ug/L) were considered.

Different studies have evaluated the utility of PCT in patients with febrile neutropenia [39-41]. A recent systematic review found 30 articles on the topic and concluded that PCT has value as a diagnostic and prognostic tool in patients with febrile neutropenia, but that due to differences in patient populations and study qualities, further research is needed [40]. Importantly in this regard, the production of PCT seems not to be attenuated by corticosteroids [42,43] and PCT production does not rely on white blood cells [44-46]. A study including 102 critically ill patients with systemic infections in a medical intensive care unit (ICU) found significantly lower CRP and IL-6 levels, but similar PCT levels, in patients treated with systemic corticosteroids (20 to 1500 mg/day of prednisone parenterally) compared to untreated patients[42]. These observations were confirmed in healthy male volunteers who received different doses of prednisolone up to 30 mg before a sepsis-like syndrome was induced with *Escherichia coli *lipopolysaccharide (LPS) injections[43]. While other biomarkers were significantly inhibited in a dose-dependent way, levels of PCT showed no inhibition within the study period. Whether this is also true for other corticosteroid doses, however, remains unknown. The value of PCT in febrile neutropenia may be as part of a combination with other biomarkers of bacterial infection such as IL-6 and IL-8 as shown in a small study of pediatric patients with febrile neutropenia [39].

## Procalcitonin as a guide for antibiotic decisions: results from randomized-controlled studies

The clinical implications of the above mentioned observational studies may be limited by differences in disease definitions and patient groups, use of insensitive (semi-quantitative) PCT assays, and different methodological issues such as observer bias, selection bias and issues of sample availability, co-infection and colonization. To overcome these limitations, several randomized-controlled studies have investigated the use of PCT to assist in decisions about initiation and/or duration of antibiotic therapy (antibiotic stewardship). Thereby the benefit of PCT was measured by clinical outcomes, assuming that if the patient recovers without antibiotics, there was no relevant bacterial illness in need of antibiotic therapy. Importantly, all intervention studies used fully automated highly sensitive PCT assays, the results of which can be obtained in the clinical routine of an emergency department within one hour thus permitting bed-side decision making. Recently, different options for PCT testing have become available, including the KRYPTOR [25], the VIDAS system (Biomerieux) [47], the Liaison BRAHMS PCT (DiaSorin)[48] and the Elecsys BRAHMS PCT (Roche Diagnostics) [49].

**Figure 2:**
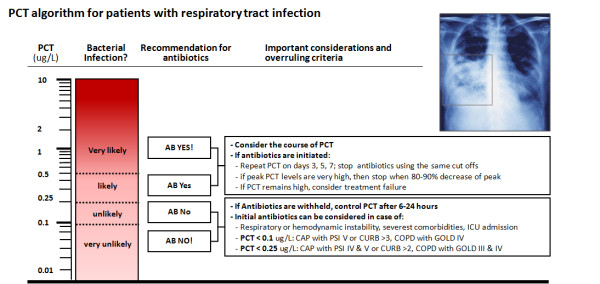
**PCT algorithm in patients with respiratory tract infections in the Emergency Department**. The clinical algorithm for antibiotic stewardship in patients with respiratory tract infections in the Emergency Department encourages (>0.5 μg/l or >0.25 μg/l) or discourages (<0.1 μg/l or <0.25 μg/l) initiation or continuation of antibiotic therapy more or less based on PCT specific cut-off ranges. Abbreviations: AB, antibiotic; LRTI, lower respiratory tract infection; PCT, procalcitonin; PSI, Pneumonia Severity Score.

**Figure 3:**
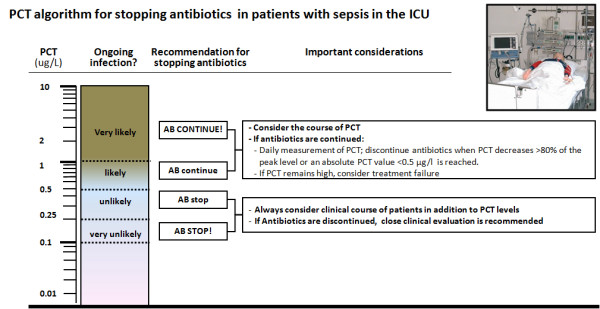
**PCT algorithm in patients with sepsis in the ICU**. In critically ill patients in the ICU, cut-offs are higher and initial empiric antibiotic therapy should be encouraged in all patients with suspicion of sepsis. PCT cut-offs are helpful in the subsequent days after admission to shorten the courses of antibiotic therapy in patients with clinical improvement. Abbreviations: AB, antibiotic; PCT, procalcitonin.

All published studies on antibiotic stewardship used similar clinical algorithms with recommendations for or against antibiotic treatment based on PCT cut-off ranges. For moderate risk patients with respiratory tract infections in the emergency department (Figure 2), algorithms recommended initiation and discontinuation of antibiotic therapy based on four different cut-off ranges. Initial antibiotics were withheld mostly in patients with low risk for systemic infection with acute bronchitis or exacerbation of chronic obstructive pulmonary disease [ECOPD]). Clinical re-evaluation and a repeated measurement of PCT were recommended after 6 to 24 hours if the clinical condition did not improve spontaneously. If PCT values were increased and antibiotic therapy was initiated, repeated PCT measurements were recommended every one to two days, depending on the clinical severity of disease, and antibiotics were discontinued using the same cut off ranges or a marked drop by 80% to 90% if initial levels were high (for example >5 μg/l). To assure safety, specific criteria were predefined where this algorithm could be overruled, such as life-threatening disease or immediate need for ICU admission. For high risk patients in the ICU setting (Figure 3), algorithms focused on discontinuation of antibiotic therapy if a patient showed a clinical recovery and PCT levels decreased to 'normal' levels, or by at least 80% to 90%.

The first intervention study testing PCT as a guide for antibiotic decisions included patients with different types and severities of respiratory infections [50]. Clinical outcomes for both groups were similar, but the PCT-guided group had markedly lower rates of antibiotic prescriptions (44% versus 83%), particularly in patients with ECOPD and acute bronchitis. Two subsequent trials evaluated the effect of PCT guidance for antibiotic discontinuation in CAP and ECOPD. PCT guidance reduced the duration of antibiotic therapy by 65% in CAP patients [16] and the prescription of antibiotics from 72% to 40% in ECOPD patients [51]. A subsequent multicenter trial [52] confirmed earlier results and found a reduction of antibiotics by 32% in CAP, by 50% in ECOPD and by 65% in acute bronchitis. Again, antibiotic exposure in ECOPD and acute bronchitis decreased mainly by not initiating treatment at all, whereas for CAP it was principally from reduction in duration of therapy. Importantly, the overall rate of adverse events was similar in both study arms and excluded a risk of more than 0.4% for PCT guided patients. Interestingly, patients with bacteremia CAP had markedly increased PCT concentrations resulting in longer treatment duration compared to culture-negative CAP patients with a lower infection-related risk [17]. Similar results in patients with respiratory infections were also reported in trials from Denmark [53] and China [54,55], and recently from an observational 'real life'- quality control survey in Switzerland [56].

Arguably, the most important (over-) use of antibiotics occurs in primary care. As many as 75% of patients with upper and lower respiratory infections receive antibiotics, despite the mostly viral origin of the condition [57]. Two multicenter primary care trials, powered for non-inferiority of clinical outcomes, investigated the safety and feasibility of PCT guided algorithms in patients with upper and lower respiratory infections, essentially relying on an initial PCT measurement only [58-60]. Both trials found substantial reductions in antibiotic exposure (by 75% and 42%), and similar clinical outcomes, particularly a similar time to recovery.

In more high risk patients in the ICU setting, different trials have investigated the use of PCT, mainly for discontinuation of antibiotics. The first small proof of concept study [61] found a four-day reduction in the duration of antibiotic therapy in patients with severe sepsis, but only in the per protocol analysis. A subsequent large multicenter trial in France recently validated this concept in more than 600 patients [62]. PCT guided patients had similar 30-day mortality rates and similar rates of relapses, but markedly more antibiotic-free days alive (14.3 versus 11.6). Another multinational ICU study focused on ventilator-associated pneumonia and found that PCT guidance resulted in a higher number of antibiotic free-days alive (13 versus 9.5 days) [63]. Two German studies assessed the effect of PCT guidance in surgical ICU patients with suspected bacterial infections in the post-operative course [64,65]. PCT guidance resulted in a significant reduction of antibiotic therapy and similar medical outcomes. In addition, the length of intensive care treatment in the PCT-guided group was significantly shorter than that in the control group (15.5 versus 17.7 days), a finding similar to the first ICU study [61]. Importantly, the use of PCT for discontinuation of antibiotics in ICU patients is still limited by a relatively small number of patients included in previous trials and awaits further large-scale validation. There are currently different ongoing trials focusing on this vulnerable patient population that should shed further light on the benefits and harms of PCT use in ICU patients.

## Limitations and areas of uncertainty

Sepsis is not a well-defined disease, but a consequence of different infectious disease entities and far too complex to be reduced to a single cut-off of any surrogate marker. Limitations of every PCT measurement include false-positive and false-negative results [8,11]. Different pathogens might induce distinct responses resulting in a variable up-regulation of circulating PCT levels [66]. While highly elevated PCT levels were found in patients with pneumococcal CAP [14], the same was not true in CAP due to atypical organisms such as mycoplasma [66]. Antimicrobial pre-treatment may influence the level of PCT resulting in lower PCT levels [67], although it remains unclear whether this relates to a direct effect or rather to lower microbial burden in patients treated with antibiotics. Unspecific elevations of PCT levels in the absence of a bacterial infection can typically be seen in situations of massive stress, for example after severe trauma and surgery [8,68-70] or in patients after cardiac shock [71]. Although the available evidence from intervention studies favors the use of PCT for de-escalation of antibiotic therapy, the same may not be true for escalation of antibiotics when PCT increases as recently demonstrated [72]. In this study PCT-guided escalation of diagnostic procedures and antibiotic therapy in the ICU did not improve survival and led to worse secondary outcomes in patients.

## Summary, future directions and conclusions

For upper and lower respiratory tract infection in ICU patients with sepsis and post-operative infections, randomized-controlled studies have shown the efficacy of using PCT algorithms to guide antibiotic decisions. For other types of infections, only observational studies are available which are importantly limited by the lack of a true gold standard. Most intervention studies were conducted in European countries including Switzerland, Germany, France and Denmark (and two in China) and validation in other countries and continents is therefore warranted. Importantly, PCT levels must always be evaluated in the context of a careful clinical and microbiological assessment. As the kinetics of PCT are of particular diagnostic and prognostic interest, repeated measurements should be performed if feasible, especially in persistently sick patients if antibiotics are withheld. The limitations of every PCT measurement include false-positive and false-negative results [8]. Unspecific elevations of PCT levels in the absence of a bacterial infection can typically be seen in situations of massive cell death, for example after severe trauma or surgery [8,68,69]. In these situations, PCT values are usually only moderately elevated and show a rapid decline in follow-up measurements. Conversely, falsely low PCT levels, typically seen during the early course or localized state of an infection, often show an increase in the follow-up measurements. Therefore, highly sensitive PCT assays are required, as subtle changes of PCT at very low concentrations can be monitored, increasing the sensitivity of the test and thus the safety of patients.

Emerging bacterial resistance to antimicrobial agents calls for more effective efforts to reduce the unnecessary and prolonged use of antibiotics in self-limiting non-bacterial and resolving diseases [1]. Patients and physicians share a common goal of improving symptoms from infection as fast as possible and often see antibiotics as the most expeditious intervention to achieve it. This one-size fits-all approach fails to consider the basic questions of who benefits from antibiotic therapy, and if treated, what would be the optimal duration. Using PCT, which mirrors the likelihood of bacterial infection and the severity of infection, to guide antibiotic therapy, is a persuasive, evidence-based approach to a more rational use of antibiotics.

## List of abbreviations

AB: antibiotic; CAP: community-acquired pneumonia; CRP: C-reactive protein; ECOPD: exacerbation of chronic obstructive pulmonary disease; ED: Emergency department; ICU: intensive care unit; IFN: interferon; IL: interleukin; LPS: lipopolysaccharide; PCT: procalcitonin; TNF: tumor necrosis factor; RCT: randomised-controlled trial; UTI: urinary tract infection; WBC: white blood cells.

## Competing interests

Drs Schuetz, Albrich, and Mueller reported receiving support from BRAHMS Inc and Biomerieux to attend meetings and fulfill speaking engagements. Dr Mueller reported serving as a consultant and receiving research support from BRAHMS and BioMérieux Inc.

## Authors' contributions

PS drafted the initial manuscript. WCA and BM commented on the manuscript. All authors approved the final version.

## Pre-publication history

The pre-publication history for this paper can be accessed here:

http://www.biomedcentral.com/1741-7015/9/107/prepub
